# Tonal cues modulate line bisection performance: preliminary evidence for a new rehabilitation prospect?

**DOI:** 10.3389/fpsyg.2013.00704

**Published:** 2013-10-07

**Authors:** Masami Ishihara, Patrice Revol, Sophie Jacquin-Courtois, Romaine Mayet, Gilles Rode, Dominique Boisson, Alessandro Farnè, Yves Rossetti

**Affiliations:** ^1^Department of Psychology, Tokyo Metropolitan University, HachiojiTokyo, Japan; ^2^Lyon Neuroscience Research Center, Institut National de la Santé Et de la Recherche Médicale, Centre National de la Recherche Scientifique, Université Claude Bernard Lyon, Hospices Civils de LyonBron, France; ^3^Services de Rééducation Neurologique, Hôpital Henry Gabrielle, Hospices Civils de Lyon, Route de VourlesSt. Genis Laval, France

**Keywords:** unilateral neglect, line bisection, pitch perception, space representation, human, rehabilitation

## Abstract

The effect of the presentation of two different auditory pitches (high and low) on manual line-bisection performance was studied to investigate the relationship between space and magnitude representations underlying motor acts. Participants were asked to mark the midpoint of a given line with a pen while they were listening a pitch via headphones. In healthy participants, the effect of the presentation order (blocked or alternative way) of auditory stimuli was tested (Experiment 1). The results showed no biasing effect of pitch in blocked-order presentation, whereas the alternative presentation modulated the line-bisection. Lower pitch produced leftward or downward bisection biases whereas higher pitch produced rightward or upward biases, suggesting that visuomotor processing can be spatially modulated by irrelevant auditory cues. In Experiment 2, the effect of such alternative stimulations in line bisection in right brain damaged patients with a unilateral neglect and without a neglect was tested. Similar biasing effects caused by auditory cues were observed although the white noise presentation also affected the patient's performance. Additionally, the effect of pitch difference was larger for the neglect patient than for the no-neglect patient as well as for healthy participants. The neglect patient's bisection performance gradually improved during the experiment and was maintained even after 1 week. It is therefore, concluded that auditory cues, characterized by both the pitch difference and the dynamic alternation, influence spatial representations. The larger biasing effect seen in the neglect patient compared to the no-neglect patient and healthy participants suggests that auditory cues could modulate the direction of the attentional bias that is characteristic of neglect patients. Thus, the alternative presentation of auditory cues could be used as rehabilitation for neglect patients. The space-pitch associations are discussed in terms of a generalized magnitude system.

## Introduction

Unilateral left spatial neglect caused by injuries to the right parietal cortex is a neurological disorder that leads to various deficits of functions such as perception, attention, representation, and action (Mesulam, 1981; Hodgson and Kennard, 2000; Milner and McIntosh, 2005). Patients who show such neglects are handicapped by poor reliability and stability of behavior in their daily lives. Such unilateral neglect patients ignore their left hemispace; “hemispace” represents either the lateral half of the space as viewed from the midline of the body (i.e., body-based hemispace) or the lateral half of the space of a fixated on object (i.e., object-based hemispace). A crucial problem underlying the disorder is the fact that patients do not question the completeness of the scene they are “seeing” because their internal representation is the whole world for them (Ishiai et al., 1987, 1989; Kinsella et al., 1993; Ishiai et al., 2006; McIntosh, 2006). Therefore, most unilateral neglect patients do not consider themselves as having any neglect (i.e., anosognosia). Possible rehabilitative treatments for neglect are; for example, caloric stimulation (Rubens, 1985; Rode and Perenin, 1994), neck vibration (Karnath, 1994; Schindler et al., 2002), limb activation (Robertson, 1991; Reinhart et al., 2012), or optokinetic stimulation (Kerkhoff et al., 2006). However, the effects of such treatments are generally transitory and last no more than 10–12 min (Rode et al., 2003; review: Rossetti and Rode, 2002; Luauté et al., 2006a). These effects are clear evidence of how simple “bottom-up” mechanisms can (albeit briefly) overcome high level cognitive deficits. Other bottom-up interventions involving vision are techniques of using Fresnel prisms (Rossi et al., 1990), eye patching (Butter and Kirsch, 1992), and prism adaptation (Rossetti et al., 1998; Frassinetti et al., 2002; Rode et al., 2003, 2006a,b; Rode and Perenin, 1994; Pisella et al., 2006; Jacquin-Courtois et al., 2013). Prism adaptation is one of the interventions that takes advantage of the effect of visuo-motor adaptation. The beneficial effect of prism adaptation on the clinical presentation of left neglect derives from the modulation of the cortical regions implicated in spatial cognition. Thus, the patients' damage to the right hemisphere may reduce its inhibitory function on the contralateral (left) hemisphere, leading to over activation of the contralateral hemisphere. This provides additional inhibition to the damaged (right) hemisphere, resulting in left neglect. Rehabilitation for such neglects, has also led to experimental manipulations using attentional orienting, such as attention training (Sohlberg and Mateer, 1987; Robertson et al., 1995) and exploration training (Kerkhoff, 1998; Keller and Lefin-Rank, 2010). These have been used in order to gain balanced hemispheric functions. Recently, theta burst stimulation (Cazzoli et al., 2009; Bonnì et al., 2013), has been developed, in such a way that the interhemispheric balance of overt attention was achieved. For the long-term functional improvement of the neglect, Luauté et al. (2006b) suggested that sensory stimulations alone do not appear to be functionally relevant to improvement, however, the coupling of both sensory and motor processing would be potentially relevant to functional improvement of neglect.

It has been shown that perceptual and motor functions in the part of the space where attention is actively directed are facilitated and that, at the same time, attention tends to be directed toward that part of the space where such functions are taking place (Tipper et al., 1992, 1998; Rizzolatti et al., 1994; Schneider and Deubel, 2002; Humphreys et al., 2004). Deficits observed with visuospatial tasks (e.g., line-bisection, line-cancellation, and picture drawing) of left neglect patients are thought to be due to redundant attentional bias toward either the right hemi space of the body or the object (Pisella and Mattingley, 2004).

With respect to the attentional bias in space of normal subjects, it has been shown that mental representations of numbers (i.e., *mental number line*) tend to modulate line bisection performance (Fischer, 2001; Calabria and Rossetti, 2005). The “*mental number line*” is a notion that describes the cognitive representation of the magnitude of numbers. The mental number line represents spatial positional codes with smaller digits (e.g., 0 or 1) on the left and larger digits (e.g., 8 or 9) on the right (e.g., Dehaene et al., 1993; Bachtold et al., Bachtold, Baumuller and Brugger; Fias, 2001; Fischer, 2001, 2003; Ito and Hatta, 2004; Ishihara et al., 2006). For example, bisections made with a pencil were biased to the left of center for a set of long strings of uniform digits, e.g., 2222 … 222 when the line was composed of smaller digits (e.g., 1 or 2) compared to the larger digits (e.g., 8 or 9) (Fischer, 2001). The same effect was also found when the stimulus line was composed of letter strings composed of number names (e.g., DEUXDEUX … or NEUFNEUF … which stands for TWOTWO … and NINENINE … in French) (Calabria and Rossetti, 2005). These results imply that the cognitive representation of the meaning of numbers (i.e., mental number line) is tightly linked to attentional and visuospatial processes, but this idea is increasingly challenged by clinical data (see Rossetti et al., 2011; Aiello et al., 2012).

Similarly to the effect of space-number association, the attentional bias in space can be modulated by the presentation of auditory pitch (Pratt, 1930; Roffler and Butler, 1968). Pratt (1930) measured the perceived location of five different pitches (in octave steps from lower to higher) in a vertical scale from the floor to the ceiling (2.5 m in height, numbered from 1 to 15). Participants, seated in front of the scale at a distance of 3 m, were asked to locate one of the numbers on the scale for the perceived position of a given tone coming from an auditory device. The results clearly showed that lower pitches were judged lower in space (the mean value was below 7) whereas higher pitches were judged in higher space (the mean was above 10), suggesting the existence of space-pitch representation along a vertical axis. This spatial effect of auditory cues (i.e., a sort of “*mental pitch line*”) in motor responses has also been demonstrated by various researchers (Mudd, 1963; Golay et al., 2005; Stevens and Arieh, 2005; Keller and Koch, 2006; Rusconi et al., 2006; Lidji et al., 2007). Rusconi et al. (2006) showed the Spatial-Musical Association of Response Codes (SMARC) effect using computer keyboard responses to auditory pitches, where higher pitches favor spatially upper responses and lower pitches favor spatially lower responses (in the reaction time analyses). Interestingly, the presence of musical expertise also showed spatial preferences of pitch (i.e., the SMARC effect) on a horizontal alignment of responses such that higher pitches favor right-side responses and lower pitches favor left-side responses (in the error analyses). These results suggest that our cognitive system maps auditory pitches onto a mental representation of space.

The effect of auditory cues also appears to differ between unilateral neglect patients and normal control participants. Golay et al. (2005) used dynamic auditory cues which are perceived as a sound moving from the right to the left ear or vice versa and measured RTs to a visual target presented at one of four horizontal locations after the brief presentation of the cue. They showed that in neglect patients RTs for left targets following the dynamic cue when moving from right to left were faster than those for the cue when moving from left to right. Static unilateral cues (i.e., a continuous tone presented either to the left or to the right ear) also modulated the visual attention of patients (i.e., generating a left advantage when following the left-ear static cue) but the degree of modulation was less than with the dynamic cues. Interestingly, such a cueing effect was not observed in normal controls. These results suggest that such auditory cues enhance the visual detection of stimuli on the impaired side of the space in neglect patients. Robertson et al. (1998) reported that in neglect patients phasic alerting by warning tones specifically accelerated the perceptual processing of left visual events, relative to right events, and that such alerting effectively produced a brief leftwards shift in spatial attention. Importantly, their results showed that the presentation of warning tones, regardless of their location, can also have a beneficial, though very transient, spatial effect in neglect patients. That is, the presentation of the alerting tones from a loudspeaker hidden at the far right of the visual screen still benefited the left visual event (Robertson et al., 1998).

As discussed above, in normal participants, visual attention can be modulated by the brief presentation of auditory cues as well as by the presentation of numeric stimuli. The difference between lower and higher pitches seems to yield spatial preferences (vertically and horizontally) in motor action. Additionally, the dynamic alternation (with a sound moving from the right to the left ear) rather than static unilateral cueing of auditory stimuli efficiently provides the left advantage in allocation of spatial attention, especially in the case of neglect patients. However, Robertson et al. (1998) reported the location (left or right) of the cueing sound presented through earphones or loudspeakers did not seem to be a critical factor. Therefore, in the present study, it was hypothesized that spatial representations would be modulated by auditory cues which are characterized by both the pitch and the dynamic alternative presentation. Moreover, this space-auditory modulation would appear more obviously in unilateral neglect patients as compared to control patients (or normal subjects) who do not show such neglects. The test task used was the line bisection because this is a traditional neuropsychological test for investigating visuospatial performance in the patients. First, the effect of the presentation order of auditory pitches with blocked manner and in alternation was tested in normal participants (Experiment 1). Then, the effect of auditory stimulation in line bisection performance for right brain damaged patients with neglect and without neglect was tested (Experiment 2) in order to explore whether auditory stimulation effectively improves the neglect patients' attentional function in space.

## Materials and methods

### Participants

In Experiment 1, healthy participants were divided into two sub groups: eight adult subjects (5 males and 3 females, mean age = 28 years old, *SD* = 6 years) participated in the experimental condition with a blocked-manner stimulus presentation, and thirteen subjects (7 males and 6 females, mean age = 25 years old, *SD* = 5 years) participated in the experimental condition with an alternative-manner stimulus presentation. They were all right-handed with normal or corrected-to-normal vision. None reported any physical dysfunction. In Experiment 2, a unilateral neglect patient (Ms. Ch, 46 years old, female) and a right brain damaged patient without neglect (Mr. Co, control patient; 65 years old, male) were tested. Ms. Ch was a 46 years old right handed woman (Oldfield test). She had a stroke on 28/01/2005. Following a thrombosis of the right medial carotid, a cortical and subcortical ischemic lesion of the whole Sylvian territory was observed. She exhibited a right fronto-temporo-parietal lesion with symptoms of left hemiplegia, anosognosia, visual neglect assessed with cancellation tasks (Albert, stars, bells), and a spontaneous deviation for both eyes and head toward the right. She did not show any sign of apraxia, and no oculomotor deficit. Functional Independence Measure: 53/126 in March, and 67 in August; MMS: 26/30 in March 2005, and around 28 in July; Albert score: 8/40; Schenkenberg: 13 omissions/20 lines, average shift = 76%; Cube drawing: 3 lines omitted on the left; Clock drawing: OK. At the time of testing, 5 months after her infarct, the patient still exhibited left hemiplegia and unilateral neglect. Mr. Co was a 65 years old right handed man (Oldfield test). The cerebral scanner revealed a right fronto-parieto-temporal lesion due to an ischaemic accident of the sylvian artery. Neurological examination revealed that the patient exhibited left hemiplegia and a discrete visual neglect evidenced from a bisection test (Schenkenberg). In the bells test, only the extreme left bells of the sheet were omitted, while the Gainotti's drawing was successfully performed. The patient was tested 5 months after his infarct and no sign of unilateral neglect was evidenced using the same test as previously used. Both patients were right-handed but have a disability of using their left hand. All subjects were informed of the experimental procedures in advance. They remained naive about the purpose of the experiment and the hypothesis being tested. This study was conducted with the informed consent of the participants, in agreement with the local ethical committee, the French law and the Declaration of Helsinki regarding patient's rights.

### Materials

A black line (200 mm in length, 2 mm in height) drawn horizontally on the middle of a white A4 sheet (210 × 297 mm, landscape orientation) was used as a stimulus item for the line bisection task. The orientation of the A4 sheet (i.e., the line) was manipulated either in the horizontal or vertical dimension. For auditory stimulation, two auditory pure-tone pitches (Low, 110 Hz and High, 1760 Hz) were used. The loudness levels of the two pitches were equalized by referring to an equal-loudness-level contours (ISO226: 2003) (Suzuki and Takeshima, 2004). The mean level of two pitches was 50 dB. In Experiment 2, a white noise of 50 dB was also used for neutral auditory stimulation. A personal computer system (Dell, Optiplex GX270, Intel Pentium 4) was used to generate auditory stimuli and those were presented to participants through headphones.

### Procedure

The participants, who were tested individually, were seated at a table in a quiet experimental room and the stimulus sheets were presented, one by one, in the mid-sagittal plane. The experimenter, who provided the stimulus sheets, was sitting in front of the participant during the experiment. The participants were required to mark the midpoint of a given line with a pencil. Once the bisection was finished, the sheet was removed and the experimenter presented the next sheet. In each trial, from the time of stimulus sheet presentation to bisection completion, the participants were exposed to the auditory pitch which was either Low or High. Each participant completed a total of 40 trials with 10 repetitions for pitches (Low and High) for each of line orientations (Horizontal and Vertical). The presentation order of auditory stimuli was blocked (e.g., Low, Low, Low, …; High, High, High, …; or High, High, High, …; Low, Low, Low, …) and alternative (e.g., High, Low, High, Low, …; or Low, High, Low, High …) in Experiment 1. The presentation order of the orientation of the stimulus sheet was counterbalanced with half of the participants began the task in the horizontal orientation, and the other half began the task in the vertical orientation. In Experiment 2, the effect of the alternative order of presentation of the two different pitches (High and Low) with the line bisection performance (only in the horizontal orientation) was tested on patients (unilateral neglect female and the no-neglect male control). The neglect patient started with 10 bisections performed using white noise. She then performed the task with the pitch alternation of “Low, High, Low, High, … ” for the first 10 trials and “High, Low, High, Low, … ” for the second 10 trials. Then, after a week, she performed 10 bisections with the white noise again. The no-neglect patient performed the task with the reversed order of alternation. These neglect and no-neglect patients did not receive any special rehabilitation procedures. They had just been following the same routine treatments (occupational therapy and physiotherapy three times a week) throughout the testing period.

### Data analysis

The subjective midpoint marked with the pencil was compared to the actual exact midpoint for each given line. Bisection biases were measured to the nearest millimeter. For the midline deviations, negative values were categorized to the left (for horizontal lines) or downward (for vertical lines) deviations and positive values to the right (for horizontal lines) or upward (for vertical lines). For Experiment 1, a Three-Way analysis of variance (ANOVA) with a mixed design [2 (presentation order: Blocked and Alternative) × 2 (line orientation: Horizontal and Vertical) × 2 (pitch: Low and High)] were performed on the deviation data. Statistical significance was set at *p* < 0.05. For Experiment 2, a t-test was performed on the deviation data for pitch. The mean deviation of the neglect patient was also compared with that of the no-neglect patient by using an estimation of the confidence interval (95%, *mean* − 1.96 × SE < μ < mean + 1.96 × SE). In addition, other specific analyses were used as described in the text.

## Results

### Experiment 1

**Figure 1:**
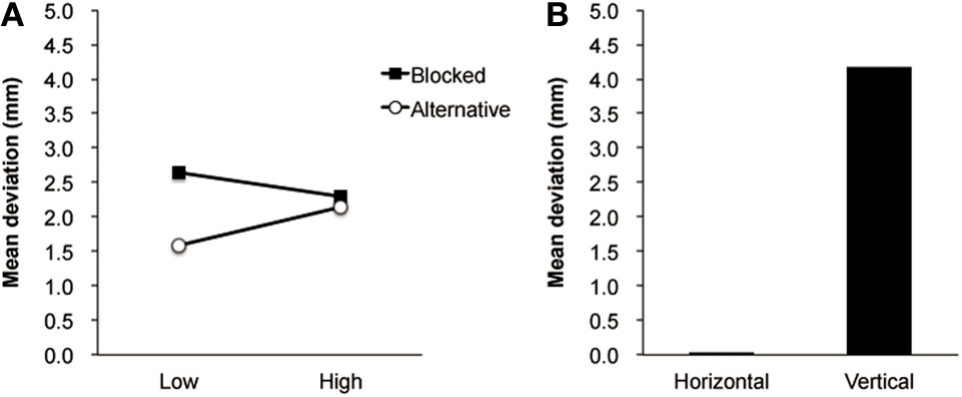
**(A)** The mean deviations of line bisection for pitch in each presentation order. **(B)** The mean deviations of line bisection for horizontal and vertical line orientation.

An ANOVA performed on the deviation data revealed a significant interaction between the presentation order and pitch factors [*F*_(1, 19)_ = 7.23, *p* < 0.05]. The analysis of simple main effects for the interaction also showed a significant simple main effect for pitch in the alternative order [*F*_(1, 19)_ = 5.36, *p* < 0.05], but not in the blocked order of stimulus presentation [*F*_(1, 19)_ = 2.22, *p* = 0.15]. The deviation of line bisection for higher pitch (2.14 mm) appeared to be larger than that for lower pitch (1.59 mm) in the alternative presentation. There were no significant simple main effects for presentation order for Low [*F*_(1, 19)_ = 1.33, *p* = 0.26] or High [*F*_(1, 19)_ = 0.03, *p* = 0.86] pitch. The mean deviations for pitch in each presentation order are shown in Figure 1A. There was a significant main effect for line orientation [*F*_(1, 19)_ = 24.34, *p* < 0.01]. The deviation of line bisection for the vertical line (4.16 mm) appeared to be larger than that for the horizontal line (0.03 mm). The mean deviations for line orientation are shown in Figure 1B^1^. Additionally, separate analyses revealed that there is a significant main effect for pitch for the horizontal line [*F*_(1, 12)_ = 10.37, *p* < 0.01], whereas there is no significant main effect for pitch for the vertical line.

### Experiment 2

**Figure 2:**
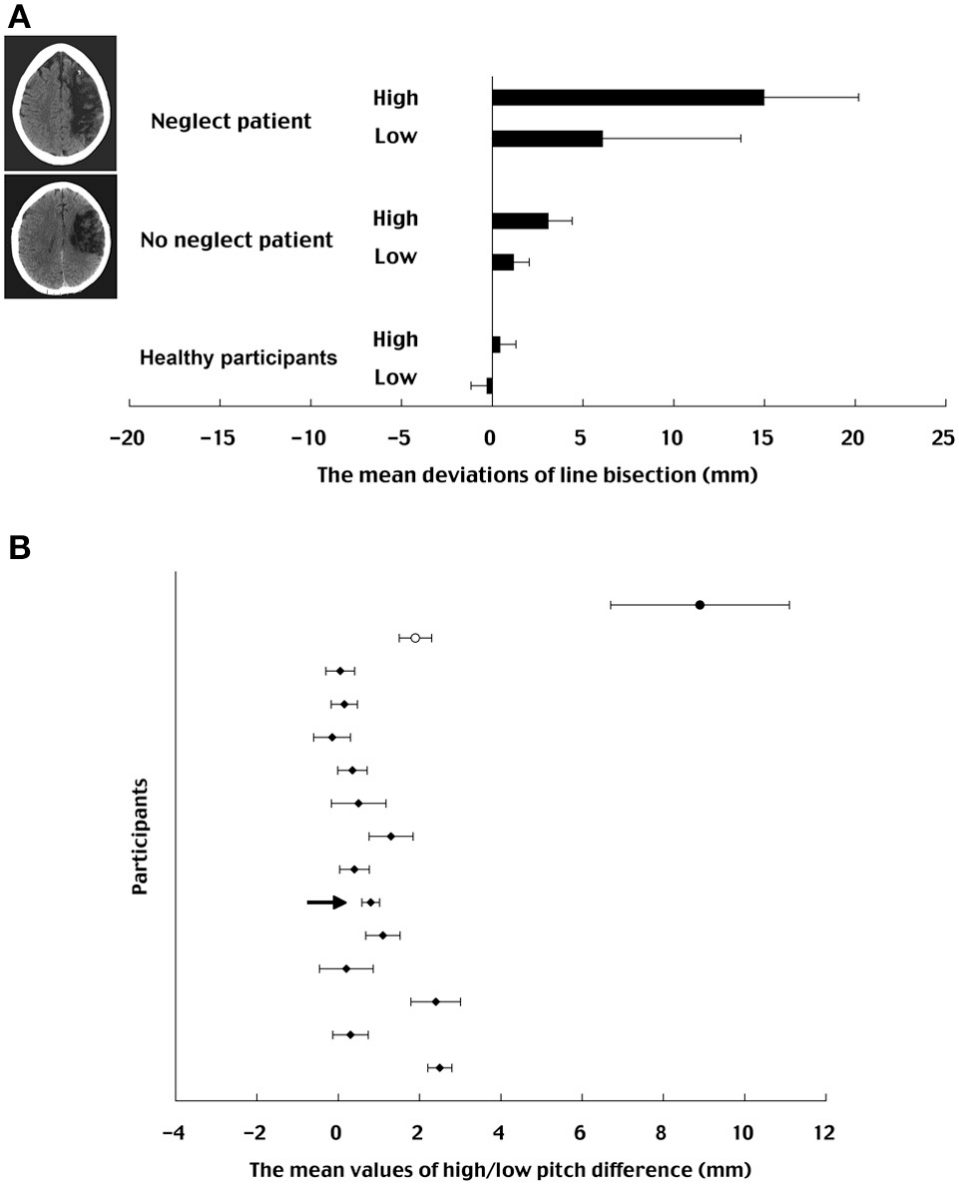
**(A)** The mean deviations of line bisection for auditory pitch in each patient. MRI scans of the neglect and no-neglect patients are presented together. The mean deviations for each pitch in healthy participants of Experiment 1 (horizontal orientation with alternative pitch presentation condition) were also imposed on the figure. Each bar indicates the range of one standard error. **(B)** The mean values of high/low pitch difference in each participant (filled circle: neglect patient; unfilled circle: no-neglect patient; filled diamond: normal healthy participants). Each bar indicates the standard deviation. The arrow indicates the data point of a selected healthy participant used for specific analyses specified in the text.

The mean deviations for auditory pitch for the two patients are shown in Figure 2A. Similar to the result obtained with normal participants in Experiment 1, the neglect patient showed a biased bisection with respect to the auditory pitch. The performance was biased to the left (6.1 mm) for the lower pitch and biased to the right (15 mm) for the higher pitch (*t* = 2.07, *df* = 9, *p* < 0.05). For the control (i.e., no neglect) patient, the difference in pitch manipulations with respect to the line bisection performance (lower pitch condition = 1.2 mm, higher pitch condition = 3.1 mm) did not reach to the significance (*t* = 1.28, *df* = 9, *p* = 0.12). The mean deviations for each pitch in healthy participants of Experiment 1 (*n* = 13, horizontal orientation with alternative pitch presentation condition) were added at the bottom of Figure 2A. The performance was biased leftward (−0.3 mm) for lower pitch and it was biased rightward (0.4 mm) for higher pitch [*F*_(1, 12)_ = 10.37, *p* < 0.01]^2^.

The deviation of line bisection in the neglect patient appeared to be much larger than that in the no-neglect patient although the variability of the neglect patient was quite large^3^. The mean bias of the neglect patient's bisection was 10.6 mm. The value of the upper confidence limits of no-neglect patient was 3.7 mm. This indicates that the line bisection performances between the neglect and no-neglect patients were significantly different from each other with deviations being larger for the neglect than for the no-neglect. Deviation data of line bisection obtained from these patients were also contrasted to those from healthy participants of Experiment 1. The value of lower confidence limits in the no-neglect patient was 0.6 mm. The mean value of healthy participants' bisection of 0.1 mm is slightly apart from the confidence interval, indicating that the no-neglect patient showed a little rightward bias as compared to healthy participants.

**Figure 3:**
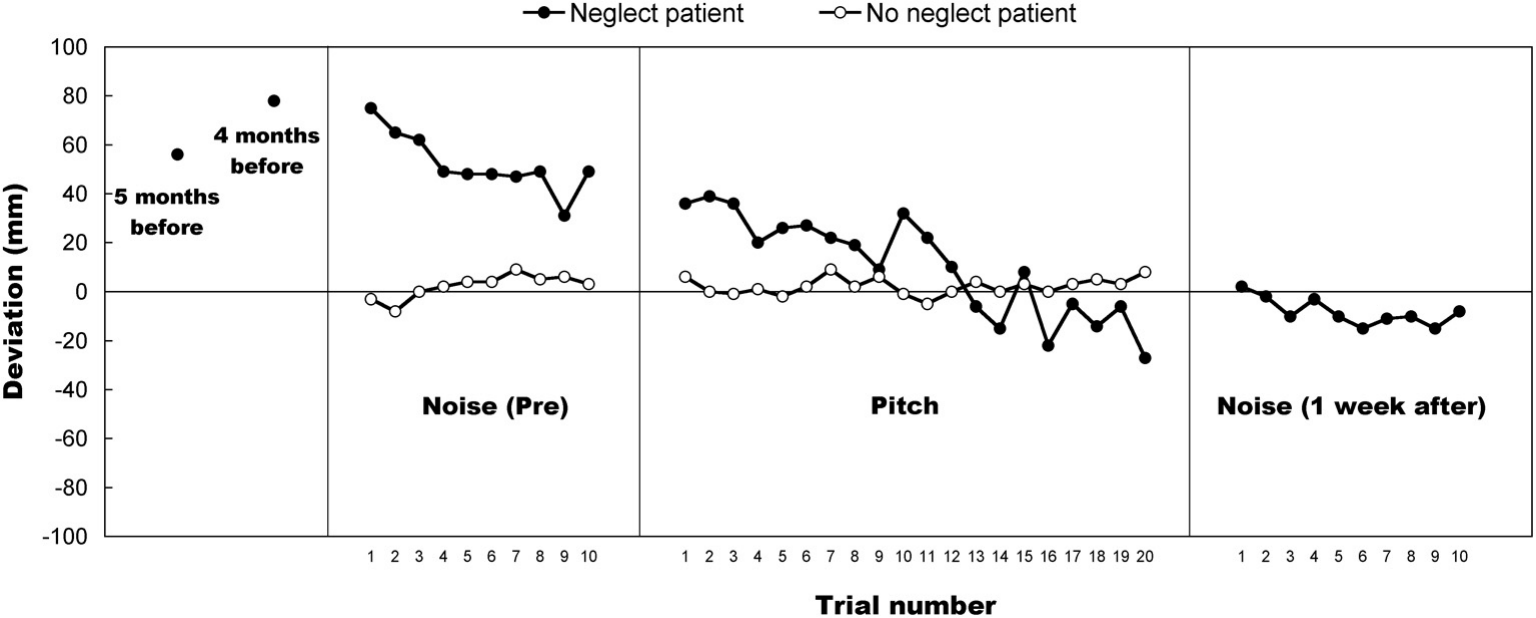
**A shift of the deviation as a function of the trial in each patient (filled circle: neglect; unfilled circle: no neglect).** The patients started with 10 bisections performed with a white noise. During this period a transient trend was observed in both patients, followed by a plateau. The neglect patient then performed the task with the pitch alternation of “Low, High, Low, High, … ” for the first half 10 trials and “High, Low, High, Low, … ” for the later half 10 trials. The no-neglect patient performed the task with the reversed order of alternation: “High, Low, High, Low, … ” for the first half 10 trials and “Low, High, Low, High, … ” for the later half 10 trials. During this period the no-neglect patient exhibited a stable performance while the neglect patient showed a sustained improvement of her bisection bias what turned into an over-compensation, i.e., leftward bisection bias. Then, after a week, the neglect patient performed 10 bisections with the white noise again, showing a sustained effect in the neglect patient. The mean deviations of line bisection (standard neuropsychological test without auditory stimulation, horizontal orientation, 200 mm in length) in the neglect patient in 4 and 5 months before the experiment are also plotted.

As is shown in Figure 2A, the effect of auditory stimulation with either higher or lower pitch in the neglect patient appeared to be much larger than in the no-neglect patient and in healthy participants. The mean value of high/low pitch difference (i.e., the size of pitch effect) in the patients was compared to that in the controls. For each patient, the differences of deviation data were calculated by subtracting the data set of lower pitch condition from that of the higher pitch condition, whereby each data set consisted of 10 bisection data in random order (i.e., permutation methods, Efron and Tibshirani, 1993). The motivation of using such a bootstrapping procedure was to examine the effect of auditory stimulation of either higher or lower pitch in each participants' category, which are the neglect patient, no-neglect patient, and the healthy participants, respectively. Since the possible combinations of calculating the differences of deviation data between the high and low pitch conditions are theoretically 100, we needed to simulate such calculations at least 100 times for each category so that the size of pitch effect could be compared to each other over the simulated sampling distributions. Therefore, the subtracting procedure was repeated 100 times and then the collected samples (10 × 100) were averaged (neglect patient: μ = 8.9 mm, σ = 2.2 mm; no-neglect patient: μ = 1.9 mm, σ = 0.4 mm). The same procedure was also applied to each healthy participant in Experiment 1 (horizontal orientation with alternative pitch presentation condition). The results are shown in Figure 2B. An ANOVA performed on the mean values of high/low pitch difference with the neglect patient, no-neglect patient, and a selected healthy participant who is nearest to the mean value of healthy participants (μ = 0.8 mm, σ = 0.9 mm) showed a significant main effect for participants' category [*F*_(2, 27)_ = 117.57, *p* < 0.01]. *Post-hoc* comparisons using Tukey's HSD tests showed significant differences between the neglect and no-neglect patients and between the neglect patient and the selected healthy participant (*p* < 0.01). There was no significant difference between the no-neglect patient and the healthy participant. The mean of high/low pitch difference for the neglect patient was significantly larger than for the no-neglect patient and for the healthy participant, showing that the size of the pitch effect appeared to be larger for the unilateral neglect patient than for the no-neglect patient or for the healthy participants.

Figure 3 depicts the evolution of bisection deviation as a function of the trial in each patient. The mean deviations of line bisection (standard neuropsychological test without auditory stimulation, horizontal orientation, 200 mm in length) in the neglect patient tested 4 and 5 months before the experiments reported here are also plotted, showing a resistant bisection bias. The neglect patient's performance gradually improved during the experimental session^4^ and was fully maintained over the post-test (a week after the experiment) performed with the white noise: the patient did not exhibit a significant bias even after 1 week post-intervention. On the other hand, the no-neglect patient did not show such an improvement across the total trials although his bisection performance was biased by the presentation of auditory pitch as explained above. To clarify the effect of auditory cues underlying the improvement seen in the neglect patient, an attempted was made to analyze the deviation data by using linear regression. First, the deviation data of line bisection for each patient were divided into three blocks, these were “Noise (pre, from 1st to 10th trial),” “Pitch 1st (the first half, from 1st to 10th trial),” and “Pitch 2nd (the second half, from 11th to 20th trial),” respectively. Then the regression was applied to the deviation data for each of the 10 trials. If the shifting pattern of slope coefficients across the three blocks was constant, the effect of auditory cues, irrespective of Noise or Pitch, can be thought to be qualitatively identical. The resulted regression lines for the neglect patient were: *y* = −3.3x + 70.4 (Noise); *y* = −2.0x + 37.4 (Pitch 1st); *y* = −3.6x + 14.3 (Pitch 2nd). The regression lines for the no-neglect patient were: *y* = 1.2x − 4.4 (Noise); *y* = 0.1x + 1.5 (Pitch 1st); *y* = 0.9x − 2.9 (Pitch 2nd). For the neglect patient, the slope of the regression lines was negative as well as higher for Noise and Pitch 2nd compared to the other block of Pitch 1st. For the no-neglect patient, on the other hand, the slope was positive and higher for Noise and Pitch 2nd compared to the other block of Pitch 1st. These results showed that in both the neglect and no-neglect patients, the slope values did not vary in a regular manner although plus/minus signs were constant throughout the blocks. Taken together, the effect of auditory cues (i.e., Noise and Pitch) in terms of the slope does not seem to be mediated by the “carry-over” influence of Noise presentation alone, but rather by independent influences of either Noise or Pitch. Additionally, the effect of the auditory cues in the neglect patient seems to be different from that in the no-neglect patient.

To further substantiate these observations, an analysis was carried out using the deviation data of line bisection in the following way. An ANOVA with a mixed design [2 (patient: neglect and no-neglect) × 3 (block: Noise, Pitch 1st, and Pitch 2nd)] was performed on these deviation data and revealed significant main effects for the patient [*F*_(1, 18)_ = 42.67, *p* < 0.01] and block [*F*_(2, 36)_ = 101.80, *p* < 0.01] factors. A significant interaction between these two factors was also observed [*F*_(2, 36)_ = 101.08, *p* < 0.01]. The analysis of simple main effects for the interaction showed significant simple main effect for the patient factor in Noise [*F*_(1, 18)_ = 147.29, *p* < 0.01] and Pitch 1st [*F*_(1, 18)_ = 58.75, *p* < 0.01], but not in Pitch 2nd [*F*_(1, 18)_ = 2.37, *p* = 0.14]. This indicates that the mean deviation of line bisection for the neglect patient was larger than that for the no-neglect patient in the first two blocks (i.e., Noise and Pitch 1st) but no difference between patients in the last block (i.e., Pitch 2nd), suggesting that the neglect was reduced at the last phase of stimulus presentation and her performance approached to the control level. Simple main effect for the block factor in the neglect patient was also significant [*F*_(2, 36)_ = 202.87, *p* < 0.01], whereas no significant simple main effect for the block factor in the no-neglect patient [*F*_(2, 36)_ =0.00, *p* = n.s.]. Multiple comparisons showed significant difference (*p* < 0.05) in all combinations of blocks. These results indicate that the mean deviation of line bisection for the neglect patient decreased with the progress of stimulus presentation [Noise (52.3 mm) \to Pitch 1st (26.6 mm) \to Pitch 2nd (−5.5 mm)], but this was not the case in the no-neglect patient. The neglect patient seemed to be more susceptible to auditory cueing compared to the no-neglect patient. Additionally, the gain in stimulus presentation on bisection performance was also calculated in order to elucidate the effect of auditory cues underlying the improvement in the neglect patient. The gain was calculated by subtracting the deviation of Noise from the deviation of Pitch 1st and was found to be −25.7 mm. The gain calculated by subtracting the deviation of Pitch 1st from the deviation of Pitch 2nd was −32.1 mm. Therefore, the influence of tonal cueing was larger compared to that of noise presentation, suggesting that the effect of Noise/Pitch on bisection performance could not be, at least, identical. Although it is difficult to totally exclude the possibility that practice had an effect on the observed deviation data, it seems plausible that the improvement in bisection performance in the neglect patient was caused by the presentation of alternating tones.

## Discussion and conclusion

The goal of this study was to test whether the presentation of auditory pitch (high or low) modulates line bisection performance. This was intended as an exploration of the interaction between space and pitch processing. The deviation of line bisection for the vertical line was larger than for the horizontal line, irrespective of high/low pitch and of blocked/alternative presentation (i.e., the effect of line orientation, see Figure 1B). This is probably reflecting the existence of a generalized magnitude system on which continuous variables are spatially plotted (Walsh, 2003), such that interactions can take place between these variables, as has been shown for example between space and auditory pitch (Rusconi et al., 2006; Lidji et al., 2007) and between space and number (Ito and Hatta, 2004) on the vertical axis. In those space-pitch and space-number experiments, using alternated or randomized presentations of numbers affected the spatially oriented behavior. Here in the present study, the alternative presentation of auditory pitches modulated the line bisection performance. Lower pitch gave rise to leftward or downward bisection biases whereas higher pitch gave rightward or upward biases, whereas there was no biasing effect of auditory pitch in blocked-order presentation (Experiment 1, Figure 1A), suggesting the interference effect resulted from a pitch contrast. This biasing effect was also observed in patients, particularly in the neglect patient with right parietal lesions (Experiment 2, Figure 2A). Furthermore, the result revealed that the effect of high/low pitch difference (i.e., the size of pitch effect) appeared to be larger for the unilateral neglect patient than for the no-neglect patient and for healthy participants (Experiment 2, Figure 2B). Unexpectedly the bisection performance of the neglect patient improved during the testing session and this improvement was still preserved even after 1 week (Experiment 2, Figure 3). These main results are discussed as follows.

First, with respect to the presentation order of auditory stimuli, the biasing effect, such as the leftward or downward bisecting with lower pitch and rightward or upward bisecting with higher pitch, was only observed in the alternative order (Experiment 1). The exposure time of the auditory stimulation in each bisection trial in both the alternative and blocked conditions was identical. However, the difference between them was whether there was an alternation of stimulus frequency, i.e., the pitch, for the next trial within one trial block. The biasing effect observed in the alternative pitch presentation (low and high) suggests a presence of spatial mechanism that is driven by the auditory presented magnitude contrast. This finding is similar to the study of number-line bisection, as the same alternating procedure was used between small and large numbers (e.g., Calabria and Rossetti, 2005), but no control block data was available.

Second, for the effect of auditory pitch in the alternative presentation, the present study demonstrated that lower pitch biased bisections to the leftward (or downward) and higher pitch biased bisections to the rightward (or upward). This tendency was the case for both the healthy participants and the neglect patient. This was similar to the biasing effect of number-line strings (Calabria and Rossetti, 2005), where bisection performance was biased by the spatial attribute of auditory stimuli as well. Such a space-pitch association is generally consistent with previous findings (Pratt, 1930; Mudd, 1963; Roffler and Butler, 1968; Golay et al., 2005; Stevens and Arieh, 2005; Keller and Koch, 2006; Rusconi et al., 2006; Lidji et al., 2007).

Third, the biasing effect in the unilateral neglect patient appeared to be larger than the patient without neglect, implying that auditory cues effectively modulated the direction of attentional bias in the neglect patient. As reported previously (Robertson et al., 1998; Golay et al., 2005), auditory cueing has a transient beneficial spatial effect in neglect patients, where the unlateralized cue enhances visual detection of stimuli on the impaired side of space. In the present experiment, the pure tone alternation improved the neglect patient's visuospatial performance. This effect might be viewed as a pseudo-lateralized stimulation derived from the alternation of low (i.e., “left”) and high (i.e., “right”) pitches that were automatically associated with spatial locations. The improvement was still preserved even after 1 week, suggesting the alternative pitch presentation could be used as a possible rehabilitative (and long-lasting) treatment for neglect patients. The space-auditory association observed in the present study using a bisection task also implies that the attentional and spatial processing could be modulated by the spatial characteristics of auditory cues. The space-pitch modulation can be thought to reflect a common level of space representation involved in the generalized magnitude system (Walsh, 2003).

One concern might be the possibility whether the biasing effect, in particular caused by higher-pitch trials that tend to produce rightward biases, could deteriorate attentional/spatial performance in the neglect patient. In this sense, one could test pitch presentations only with lower-pitch trials that presumably produce leftward biases. As is shown in the present study, however, the biasing effect of pitch on the line-bisection performance was not found in the blocked-order presentation, but was found in the alternative presentation, implying that the experimental manipulation of pitch contrasts seem to be important. Additionally, as is shown in the prism adaptation study on left neglect patients (Rossetti et al., 1998; Luauté et al., 2006b; Pisella et al., 2006; Rode et al., 2006a), prismatic lens-mounted goggles that create an optical shift to the “right” have been used for their rehabilitation. Patients exposed to such a shift of the right visual field reduced their biased perception of the body-midline (as a result of sensorimotor aftereffects) and improved their performance on classical neglect neuropsychological tests. Therefore, the rightward bias produced by higher pitch stimulation, which is akin to the rightward attentional orienting by the prismatic lens and does not seem to deteriorate the attentional/spatial performance in the neglect patient.

Although our approach could be of benefit to neglect patients, the method of stimulus presentation using tone alternations did not fully answer the question whether the “alternation” would really modulate their visuospatial performance, because we did not test the effect of two different pitch presentations in a completely random or in a semi-random way. Such a simple alternation might have driven an expectation in participants, resulting in the benefits. This discussion raises many numerous hypotheses about the meaning of stimulus presentation and directions for future research in the field of multimodal rehabilitation (Keller and Lefin-Rank, 2010; Jacobs et al., 2012). Future studies are needed that could answer the question of whether the beneficial spatial effect seen was due to the alternation with the expected attentional mechanism or due to a randomly modulated implicit mechanism.

There is another concern about the stimulus presentation. This is the effect of white noise on line bisection performance. Recently, Cattaneo et al. (2012) investigated bisection biases with a white noise in healthy individuals and showed that the noise affected their performance in both visual and haptic bisection, reducing their leftward error (i.e., a pseudoneglect in which neurologically intact individuals usually show as a manifestation of a right-hemisphere dominance in controlling the allocation of spatial attention). They interpreted that to mean that the effect would be due to noise presentation and that the noise might boost alertness and restore the hemispheric activation balance. The effect of white noise stimulation in the present study was similar to their result. Namely, that the spatially independent and task irrelevant, results in the no-neglect patient actually reduced his leftward error (i.e., pseudoneglect) with a positive slope coefficient during the session (“Noise” block in Figure 3, see the slope coefficient analyses in the result section). This result is consistent with what was found in Cattaneo et al. On the other hand, as shown in Figure 3, the bisection performance of the neglect patient gradually improved (i.e., reducing her rightward error) even when a white noise was used as a neutral auditory stimulus. Such opposite effects found in the no-neglect and neglect patients imply that auditory stimulations might have different impacts on spatial attention in these individuals, inducing a rightward shift in the no-neglect patient and a leftward shift in the neglect patient (Cattaneo et al., 2012). The results found in the present study also suggest that this effect saturated in both patients before the end of the white noise stimulation. However, since there was no condition without noise presentation in the present study, it is still difficult to totally exclude the influence of practice (i.e., test repetition) from the observed deviation data. Future research is needed to answer the question of whether the improvements would be specific for the white noise presentation or the test repetition.

Walsh (2003) has argued that the inferior parietal lobule (IPL) reflects the common need for space, time, and quantity information to be used in sensorimotor transformations, suggesting that the IPL is a generalized magnitude system for action. Impairment of the IPL (and/or the lateral temporal lobule) often causes unilateral neglect (Vallar and Perani, 1986; Driver and Mattingley, 1998; Mattingley et al., 1998; Mort et al., 2003; Golay et al., 2008). It is evident that unilateral neglect patients with right parietal lesions show deficits such as in sensorimotor and more cognitive spatial functions (Rossetti et al., 1998; Rode et al., 2001, 2003; Farne et al., 2003; Milner and McIntosh, 2005). Further, the parietal cortex has been recognized as a heart of on-line action processing (Pisella et al., 2000; Grea et al., 2002; Rossetti et al., 2005). As proposed by Walsh, the IPL can be viewed as a generalized magnitude system for action. A significantly larger biasing effect modulated by the auditory cues observed only in the neglect patient, compared to the no-neglect patient, could be explained by an IPL lesion in the first patient.

As has been discussed above, the present study demonstrated the spatial effect of auditory cues in the neglect patient. This suggests that auditory cues, which have different magnitude information, effectively modulate the direction of the attentional bias in the neglect patient. Different-pitch cues might have an implicit left-right mapping when these are transferred into spatial coordinates. Other experiments might further assist in testing this prediction; for example, by alternating the duration of the short and long tones to see if temporal perception is also altered in neglect patients (Calabria et al., 2011).

In conclusion, the present study demonstrated that auditory cues characterized by both the pitch difference and the dynamic alternation (i.e., pitch alternation) produce preferences of the line bisection performance in space. As predicted, the cueing effect in the neglect patient appeared to be larger than that in the no-neglect patient and in healthy participants, suggesting that in the neglect patient auditory cues modulate the direction of attentional bias. The biasing effect as well as the improvement of the neglect seems to be of beneficial spatial effect due to the auditory cue. This may reflect the impaired integration of multisensory spatial information in the neglect patient (Keller and Lefin-Rank, 2010; Jacobs et al., 2012). The space-auditory association in the bisection task also implies that the attentional and spatial processing can be modulated by the presentation of auditory cues. The space-pitch modulation observed in the present study can be thought to reflect the common level of space representation which is tightly linked to multisensory integration involved in the generalized magnitude system. Since the present study demonstrated the single-case results from the neglect and no-neglect patients, more research is needed to assess the multisensory conception of neglect.

### Conflict of interest statement

The authors declare that the research was conducted in the absence of any commercial or financial relationships that could be construed as a potential conflict of interest.

## Footnotes

^1^There were no other significant main effects nor interactions [presentation order: *F*_(1, 19)_ = 0.43, *p* = 0.52; pitch: *F*_(1, 19)_ = 0.34, *p* = 0.57; presentation order × line orientation: *F*_(1, 19)_ = 0.63, *p* = 0.44; line orientation × pitch: *F*_(1, 19)_ = 1.25, *p* = 0.28; presentation order × line orientation × pitch: *F*_(1, 19)_ = 0.00, *p* = 1.00.]

^2^A slight leftward bias (i.e., pseudoneglect) in healthy participants is not always observed in the line bisection performance, for example, musicians tend to show no pseudoneglect or even rightward bias (Patston et al., 2006). Many of our normal participants might have more or less musical experiences, resulting in no pseudoneglect.

^3^*F*-test performed on patients' bisection data (alternative pitch presentation condition of 20 trials) revealed a significant difference of variance between the neglect and no-neglect patients [*F*_(1, 38)_ = 38.86, *p* < 0.01]. We also detrended the data by subtracting the linear regression line applied to each patient (neglect: *y* = −3.1x + 43.1, *R*^2^ = 0.80; no neglect: *y* = 0.1x + 0.9, *R*^2^ = 0.04) and examined the variance of resulted residual scores. The result showed that there is a significant difference of variance between these patients [*F*_(1, 38)_ = 11.12, *p* < 0.01]. The large variability of line bisection in the neglect patient seems to reflect the biasing effect of alternative pitch presentation as well as the evolution of bisection performance as a function of the trial (also see the text regarding Figures 2B, 3).

^4^The linear regression line applied to the deviation data of line bisection (alternative pitch presentation condition) in the neglect patient showed a negative slope as described in Footnote 3. The mean values of both lower (6.1 mm) and higher (15 mm) pitches appeared to be much left of pre-noise (52.3 mm) stimulations, suggesting that the tonal cues were more effective (compared to the simple noise presentation) to reduce the influence of impairment (i.e., unilateral neglect) across the trials. For more details, see the slope coefficient analyses in the result section.
